# Relationships Between Identity, Well-Being, and Willingness to Sacrifice in Personal and Collective Favorite Places: The Mediating Role of Well-Being

**DOI:** 10.3389/fpsyg.2020.00151

**Published:** 2020-02-07

**Authors:** Igor Knez, Ingegärd Eliasson, Eva Gustavsson

**Affiliations:** ^1^Department of Health Science and Psychology, University of Gävle, Gävle, Sweden; ^2^Department of Conservation, University of Gothenburg, Göteborg, Sweden; ^3^Department of Conservation, University of Gothenburg, Mariestad, Sweden

**Keywords:** place identity, well-being, willingness to sacrifice, favorite places, biosphere reserve

## Abstract

In line with research indicating positive associations between well-being and personal and collective people-place bonding, and that collectivistic compared to individualistic commitment may have stronger associations with pro-environmental behavior, we investigated relationships between identity, well-being, and willingness to sacrifice (type of pro-environmental behavior) in personal and collective favorite places. A total of 884 respondents, living in three Swedish municipalities, participated in this study. In line with the hypotheses, we showed *congruent* positive relationships between place-related: (1) *personal* identity and *personal* well-being; (2) *collective* identity and *collective* well-being, (3) *collective* identity and *collective* willingness to sacrifice; and (4) an *incongruent* positive association between *collective* identity and *personal* willingness to sacrifice. Additionally, a significant role of well-being in mediating the identity → willingness to sacrifice relationship was reported, suggesting that our willingness to pay higher taxes and prices and to accept cuts in standard of living in order to protect our personal and collective favorite places might be accounted for partly by how we feel visiting these places.

## Introduction

We are not placeless (Casey, 2000). We bond as individuals and collectives to physical sites in our lives (Lewicka, 2008; Knez, 2014). We also feel good when visiting these favorite places (Knez and Eliasson, 2017; Knez et al., 2018b). We miss them when they are gone (Knez et al., 2018a), and we behave pro-environmentally to protect them implying, for example, an instrumental milieu-related value (to care for the good of a site because it satisfies our needs; see, e.g., Dietz et al., 2005 for a review). Given this, the aim of the present study was to investigate relationships between three important place-related phenomena of identity (emotional and cognitive bonds tied to personal and collective favorite places), well-being (feelings of wellness associated with personal and collective favorite places), and willingness to sacrifice (type of pro-environmental behavior ascribed to personal and collective favorite places; that is, pay higher taxes and prices and accept cuts in standard of living to protect favorite places). As far as we know, these relationships have not been addressed by previous research.

### Place-Related Identity

Humans bond with physical sites involving psychological, social, historical, cultural, and health dimensions (Knez, 2005; Lewicka, 2008; Lachowycz and Jones, 2013; Butler et al., 2019). These ties act as reminders of significant collective and personal encounters (Knez, 2006; Lewicka, 2008; Wang, 2008; Taylor, 2010; Wheeler, 2014) involving emotional, cognitive, and behavioral operations in how we perceive ourselves as individuals and collectives (Canter, 1997; Casey, 2000; Knez and Thorsson, 2006; Knez, 2014). All this results in different types of place-related identifications (Twigger-Ross et al., 2003; Wang, 2008; Stobbelaar and Pedroli, 2011; Clayton, 2012; Wheeler, 2014). In other words, we link our lives to physical places which, by this, situate our “memorial life” (Casey, 2000).

People-place bonds include both personal (Knez, 2006; Taylor, 2010) and collective (Lewicka, 2008) emotional and cognitive ties, by which we maintain and strengthen our place-related identifications (Wang, 2008; Wheeler, 2014). In addition, collective identity relates to “group membership, group processes and intergroup behavior,” while personal identity is associated with “close personal relationships and idiosyncratic attributes” (Hogg, 2006, p. 463). These experiences are apportioned across declarative memory as autobiographical memory; hence, a self-related memory (Kihlström and Klein, 1994; Conway, 2005) phenomenologically shared as a life story (Fivush, 2008). The result is a feeling of “re-living the past” (Klein, 2013, p. 3), when we communicate in spoken and written words about our lives and ourselves.

In line with the autobiographical memory approach, Knez (2014; see also Knez, 2016b) proposed a role for emotional and cognitive constituents accounting for the phenomenon of place-related identity, involving cognitive processes of coherence, correspondence, mental temporality (inner “time travel”), reflection, and agency (Conway et al., 2004; Klein et al., 2004), as well as an emotional process of attachment/closeness/belonging (Marris, 1982; Hidalgo and Hernandez, 2001; Knez, 2005; however, see Knez and Eliasson, 2017 for a more elaborated discussion about different place-attachment/identity approaches in environmental psychology). Given this, we think, remember and reason about, and feel closeness to our favorite physical sites. In the words of Knez (2014), p. 186): “…places and time *position*-anchor one’s reminiscence by forming psychological person-place ties, emotional and cognitive bonds that conduct the psychological agent toward physical place and time as *the* organizing formats for its personal memory.”

The psychological phenomenon of place-related identity also includes information about the surrounding nature (Knez, 2005, 2006; Knez and Eliasson, 2017; Butler et al., 2019), which has been reported to be associated with: (1) Curative feelings of well-being (Korpela, 1989, 1992; Korpela and Hartig, 1996; Knez, 2006) for both personal and collective favorite places (Knez and Eliasson, 2017); and (2) Emotional loss of a beloved site after a natural disaster (Knez et al., 2018a; Butler et al., 2019). Thus: “Natural or semi-natural features of the environment are often associated with the identity of an individual, a community, or a society. They provide experiences shared across generations, as well as settings for communal interactions important to cultural ties” (Daniel et al., 2012, p. 8814). Consequently, natural and cultural attributes and features of a physical site are central to the self, identity and memory (Knez, 2005, 2006; Lowenthal, 2005; Aplin, 2007; Erll, 2011; Butler et al., 2019).

### Place-Related Well-Being

For a long time, humans have related to, and bonded with, nature due to its curative and restorative dimensions (Wilson, 1984; Kellert and Wilson, 1993; Gesler, 2000). In many ancient cultures archetypical landscapes, e.g., Garden of Eden, have long been connected with supreme types of life and well-being (Ward Thompson, 2011). Also, in fiction, self-biographical nature-related reflections are portrayed, e.g., “pure Alpine air and magnificent mountain landscapes” (Gesler, 2000, p. 126) and “When I am lonely the mountains call me” (Griffin-Pierce, 1997, p. 1). The scientific investigation of the relationship between humans and the surrounding nature started, we might say, in 1732 when the eminent natural scientist, Carl von Linné suggested, based on his empirical observations, an association between nature and human well-being (Linné von and James Edward, 1811).

In view of this, empirical studies of relationships between nature and human well-being have reported many different types of associations, involving feelings of solitude, aesthetic values, sense of timelessness, positive affect, and stress reduction (e.g., Laski, 1961; Williams and Harvey, 2001; Park et al., 2011; Russell, 2012; Hedblom et al., 2019). This research comprised measures of social, psychological, and physiological variables (Abraham et al., 2010; Bowler et al., 2010; Hartig et al., 2011; Carrus et al., 2015; Sandifer et al., 2015) including both rural (e.g., Knez and Eliasson, 2017; Knez et al., 2018a; Butler et al., 2019) and urban (e.g., Carrus et al., 2015; Gunnarsson et al., 2016; Ode Sang et al., 2016; Hedblom et al., 2017; Panno et al., 2017; Knez et al., 2018b; Hedblom et al., 2019) types of greenery.

For the most part, these results have been given emotional, aesthetic, and cognitive explanations (e.g., Ulrich, 1983; Kaplan, 1995), overlooking the importance of identity, memory, and well-being links with personal and collective nature-related favorite sites. However, Ratcliffe and Korpela (2016), Knez and Eliasson (2017), and Morton et al. (2017) have recently addressed these issues. Knez and Eliasson (2017) showed, for example, a positive relationship between well-being and nature-related place identity in personal and collective favorite places, implying a mediating role for place-identity in the nature-well-being relationship.

This was recently supported by Knez et al. (2018b) showing that when visiting their favorite high-naturalness places, residents perceived higher levels of well-being. A mediation analysis additionally reported that a naturalness-well-being link was, to a certain degree, accounted for by the place-identity, especially the emotion component of people-place bonding (Knez et al., 2018b; see also Knez et al., 2018a). This suggests, theoretically, that the self, in a self-regulating way (Korpela, 1989, 1992; Korpela and Hartig, 1996; Knez, 2006, 2014), may promote processes of affect-regulation (Parkinson and Totterdell, 1999; Korpela et al., 2001), meaning that the self will enjoy the greenery of places associated with strong place-identity and by that boost the processes of well-being.

Finally, the relations between nature and place-related self/identity has additionally been measured by, for example, connectedness and connectivity to nature (Mayer and Frantz, 2004; Dutcher et al., 2007), and inclusion of nature into the self (Schultz, 2002). However, as far we know, no studies have investigated the relationships between personal and collective people-place ties and well-being in personal and collective favorite sites respectively.

### Place-Related Willingness to Sacrifice

Pro-environmental behavior as a phenomenon is defined by the activities oriented toward sustainability (Gardner and Stern, 1996; Kaiser, 1998; Di Castri, 2000; Stern, 2000; Bonnes and Bonaiuto, 2002; Steg and Vlek, 2009). Three such activities are deduced from theories of planned behavior (Ajzen, 1991), reason action (Fishbein and Ajzen, 1975; Ajzen and Fishbein, 1980) and norm activation (Schwartz, 1970, 1977); namely, behavioral control, willingness to sacrifice and action behavior (Oreg and Katz-Gerro, 2006).

Willingness to sacrifice, which is addressed in this paper, represents an environment-related *behavioral intention*. More precisely, a willingness to: (1) Pay *higher taxes* to protect the environment; (2) Pay *higher prices* to protect the environment and (3) Accept *cuts in standard* of living to protect the environment (Oreg and Katz-Gerro, 2006; Knez, 2016a). This phenomenon, in general terms, is associated with strong commitment; for example, in close relationships (Lange van et al., 1997). Previous research has indicated, for example, that people with strong egoistic values will perceive less willingness to sacrifice, and vice versa for those with strong altruistic values (Knez, 2013, 2016a). Additionally, Davis et al. (2011) have reported that commitment to natural sites (measured as place attachment) may predict willingness to sacrifice. Furthermore, Iwata (2002) and Chen and Zheng (2016) have indicated positive relationships between environmental responsibility and willingness to sacrifice.

Thus, and in the words of Davis et al. (2011, p. 3): “willingness to sacrifice for the environment represents the extent to which individuals’ decisions will take into account the well-being of the environment, even at the expense of immediate self-interest, effort, or costs.” In line with this, Brown and Kasser (2005) have shown positive associations between ecologically responsible behavior (type of pro-environmental behavior) and well-being. Several studies have also indicated relationships between commitment and pro-environmental behavioral intentions (Davis et al., 2009), tentatively suggesting that collective vs. personal commitment may operate better in an environmental perspective (Lubell, 2002; Clayton, 2003; Wakefield et al., 2006). In the words of Steg and Vlek (2009): “…the more strongly individuals subscribe to values beyond their immediate own interests, that is, self-transcendent, prosocial, altruistic or biospheric values, the more likely they are to engage in pro-environmental behavior.”

It has been suggested that classical social identity theory (Tajfel, 1978; Tajfel and Turner, 1986) may account for this type of finding. That is, a social identity, a type of social commitment, may encourage individuals to define themselves more as a group of environmentalists than individuals (Brunsting and Postmes, 2002; Opotow and Brook, 2003; Dono et al., 2010). In line with this, we will investigate how the behavioral intention of willingness to sacrifice applies to personal and collective favorite places, as types of commitment (place-identities). Both commitment and identification with a place involve the psychological process of associating the self closely with a place, implying that the self might feel part of the place. For that reason, the phenomenon of supporting and making oneself responsible for a place (commitment) can be measured with people-place bonding instruments (see section “Place Identity” for details).

### Present Study and Hypotheses

We formulated *six hypotheses* (see below) regarding relationships between place-related phenomena of identity, well-being, and willingness to sacrifice. This was done in line with previous research reporting: (1) positive associations between place-identity and well-being (e.g., Knez and Eliasson, 2017; Knez et al., 2018a, b); (2) a relationship between commitment and pro-environmental behavior (e.g., Iwata, 2002; Davis et al., 2011; Chen and Zheng, 2016); (3) a tentative advantage of collective over individual commitment in pro-environmental behavior (e.g., Brunsting and Postmes, 2002; Lubell, 2002; Clayton, 2003; Opotow and Brook, 2003; Wakefield et al., 2006; Davis et al., 2009; Dono et al., 2010); and (4) an association between well-being and pro-environmental behavior (e.g., Brown and Kasser, 2005; Davis et al., 2011).

In addition and following Knez and Eliasson (2017), *congruent* (personal-personal and collective-collective) compared to *incongruent* (personal-collective and collective-personal) relationships (hypotheses 1, 2, 3, and 4) were predicted to be stronger. On the other hand, in line with, for example, Clayton (2003) and Dono et al. (2010), findings suggesting an advantage of collective vs. individual commitment in pro-environmental behavior, *incongruent* (collective-personal) compared to *congruent* (personal-personal) relationship, hypothesis 5, were predicted to be stronger. Accordingly, we hypothesized:

(1) Positive congruent association between *personal* (vs. collective) identity and *personal* well-being.
(2) Positive congruent association between *personal* (vs. collective) identity and *personal* willingness to sacrifice.
(3) Positive congruent association between *collective* (vs. personal) identity and *collective* well-being.
(4) Positive congruent association between *collective* (vs. personal) identity and *collective* willingness to sacrifice.
(5) Incongruent positive association between *collective* identity and *personal* willingness to sacrifice.
(6) In line with research indicating positive relationships between well-being and pro-environmental behavior (e.g., Brown and Kasser, 2005; Davis et al., 2011), we also predicted that associations between identity and willingness to sacrifice might be *mediated*, to some extent, by well-being ascribed to personal and collective favorite places.

## Materials and Methods

### Study Area

**FIGURE 1:**
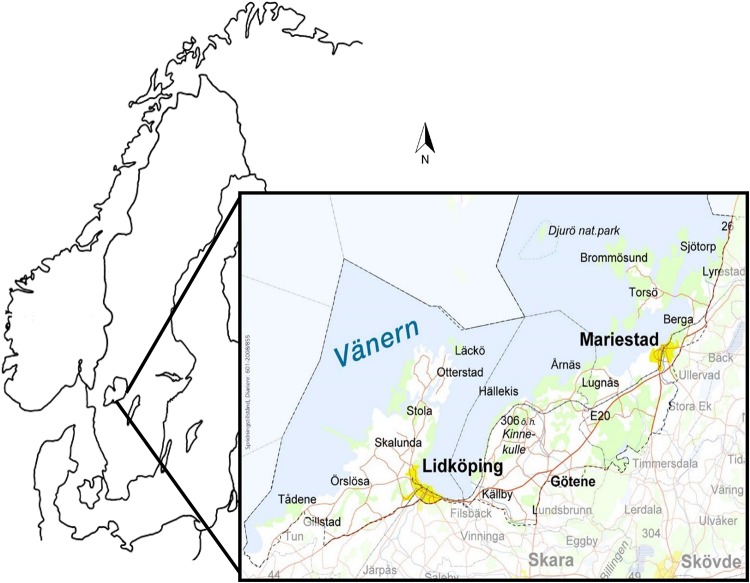
Study area consists of the municipalities Mariestad, Götene and Lidköping, hosting the depicted Biosphere Reserve Lake Vänern Kinnekulle.

The study area includes the municipalities Mariestad, Götene and Lidköping (1706 km^2^ land and 1791 km^2^ lake), where Lake Vänern Archipelago Biosphere Reserve is situated (40° E, 65° N). The area runs along the southeastern shore of Lake Vänern in the southwestern part of Sweden (see Figure 1). Much of the landscape (42%) is covered by arable land on post-glacial clayey plains, explaining the numerous food industries in this part of Sweden. Along the Vänern shoreline, gneiss ridges break up the plain and, continuing out into the water, form an island-rich archipelago in this, the largest lake in the European Union (Drotz et al., 2014). The climate is slightly maritime due to the large body of water. The average temperature in January is −3°C and in July 15°C. Mylonite intrusions, glacial moraine deposits and the Cambro-Silurian hill Kinnekulle, provide variation in the topography and bedrock chemical content; thereby creating varying prerequisites for biodiversity. People have lived in this area for at least 6,000 years (Drotz et al., 2014). An abundance of pre-historical and historical landmarks and artifacts dating back to the Bronze Age also imply millennia of cultivation and influence on the landscape, still visible in the diversity of plant species (Gustavsson et al., 2007).

### Sample

A total of 2,989 households, identified from a population register, were sent a “landscape survey.” They were randomly and proportionally distributed across three municipalities (Lidköping 51.2%, Mariestad 31.5%, and Götene 17.2%) encompassing the Lake Vänern Archipelago Biosphere Reserve. Participants were not offered any incentive to participate. The survey involved several sections, including qualitative and quantitative questions. Data on place-related values, personal and collective identity, well-being, and willingness to sacrifice are reported in this study. The survey was anonymous and was conducted in accordance with the ethical guidelines of APA and the University of Gothenburg Sweden, which was in charge of the project. Therefore, it was not reviewed and approved by any special ethic committee.

### Procedures and Demographic Statistics

Surveys were distributed and returned by mail. After four reminders (phone contacts), 884 responses (29.6%) were achieved, proportionally distributed across the three municipalities (Lidköping 50.5%, Mariestad 32.2%, and Götene 17.3%). Answers were distributed across 56.8% females and 43.1% males, and seven age groups 18–25 (2.1%), 26–36 (6.6%), 36–45 (10.4%), 46–55 (17.9%), 56–65 (17.8%), 66–75 (29.1%), and 76- (16%). Participants’ mean length of residence was 38.5 years, ranging from 1 to 87 years. Educational background was distributed across three types of education: elementary education (22.9%), upper secondary education (38.8%), and university education (34.4%). Most of the participants were employed (48.1%) or retirees (48.4%).

### Measures

#### Valuations and Categorizations of Personal and Collective Favorite Places (Qualitative Data)

In line with Knez and Eliasson (2017) we asked: *Which three places in the landscape are the most important for you personally? By this we mean places that are your favorites; places which increase your understanding of who you are?* Participants were asked to write down the names of these places or mark them on a map included in the survey. They were further asked to *select one of the three places -your favorite site-* and to write down what they valued most about this place. All this was repeated for the collective favorite places: *Which three places in the landscape are the most important for you living in the* Lake Vänern Archipelago Biosphere Reserve; *that is, sites that enhance understanding of the identity of the* Biosphere Reserve*? (Note: singular form o*f “you” in Swedish is “dig,” and plural form is “er”; consequently, connoting directly to personal vs. collective dimensions of “you”). Finally, participants were asked to categorize their personal and collective favorite places as belonging to environmental categories of archipelago, agricultural landscape, forest, built-up environment, or other.

#### Place Identity

Davis et al. (2011) measured commitment to natural sites with a place attachment instrument. We used a measure of place-identity comprising both an emotional (place-related attachment/closeness/belonging) and a cognitive (place-related coherence, correspondence, mental temporality, reflection, and agency) component of people-place bonding (Knez, 2014; Knez and Eliasson, 2017; Butler et al., 2019). This measure involves autobiographical emotional and cognitive components, comprising 10 statements (Knez and Eliasson, 2017). *Emotional component* (processes of attachment/closeness/belonging; in the present study, with a Cronbach alpha of 0.90): “I am keenly familiar with the place.” (emotional familiarity); “I miss it when I’m not there. (emotional missing)”; “I have strong ties to the place.” (emotional bond); “I am proud of the place.” (emotional pride); “The place is a part of me.” (emotional agency). *Cognitive component* (processes of coherence, correspondence, mental temporality, reflection and agency; in the present study, with a Cronbach alpha of 0.93): “I have had a personal contact with this place over a long period.” (coherence); “There is a link between the place and my current life.” (correspondence); “I can travel back and forth in time mentally to this place when I think about it.” (mental temporality); “I can reflect on the memories attached to this place.” (reflection); “These thoughts about the place are part of me.” (cognitive agency). Participants were asked to respond to these statements on a seven-point scale, ranging from 1 (completely disagree) to 7 (completely agree). For the collective place identity measure, we changed the pronoun “I” to “we (living in the **XX**).”

#### Well-Being

Participants were asked to respond to 10 statements from “The WHO (ten) well-being index” (Beach et al., 1996), measuring their place-related well-being. They responded to the question, *when I’m on the site, I feel*: “Sad and down” (R); “Calm and relaxed”; “Energetic, active and enterprising”; “Relaxed and refreshed”; “Happy and pleased with my personal life”; “Satisfied with my living situation”; “I live the life I want to live”; “Inspired to deal with today’s work”; “I can cope with serious problems or changes in my life”; “That life is full of interesting things.” Furthermore, the four-point scale from the original measure was rearranged yielding a seven-point scale, ranging from 1 (completely disagree) to 7 (completely agree), with a Cronbach alpha of 0.90.

#### Willingness to Sacrifice

This measure involved three items (Oreg and Katz-Gerro, 2006; Knez, 2016a): “I am willing to pay higher taxes to protect the environment.”; “I am willing to pay higher prices to protect the environment.”; and “I am willing to accept cuts in standard of living to protect the environment.” with Cronbach alpha of 0.91. Participants were asked to respond to these statements on a seven-point scale ranging from 1 (completely disagree) to 7 (completely agree).

### Design and Analyses

Hypotheses 1–5 were estimated with multiple regression analyses, including the two types of place identity as predictors, and well-being/willingness to sacrifice as criterion variable. Hypothesis 6, that is, the mediating role of well-being in identity → willingness to sacrifice link was investigated by performing two mediation analyses, one for each type of identity; using the plug-in PROCESS (Hayes, 2013) developed for IBM SPSS Statistics.

## Results

We first report qualitative results involving the valuations and categorizations of personal and collective favorite places, and second, quantitative results including (1) regression analyses related to the 1–5 hypotheses, and (2) mediation analyses related to the question about the mediation role of well-being in the links between identity and willingness to sacrifice (Hypothesis 6).

### Valuations and Categorizations of Personal and Collective Favorite Places

#### Personal Favorite Places

Participants categorized their personal favorite places as being Archipelago (33%); Forest (24%); Agricultural landscape (19%); Built-up environment (15%); and Other (9%). The five most valued attributes related to personal favorite places were: Natural environment 32% (nature, forest, fauna); Lake area 20% (lake, water, archipelago); Tranquility 13% (calmness, silence, freedom); View/beautiful 11%; and Home/family 9% (family, friends, socialize).

#### Collective Favorite Places

These places were categorized as being: Archipelago (23%); Forest (20%); Built-up Environment (20%); Agricultural landscape (19%); and Other (9%). The five most valued attributes related to collective favorite places were: Natural environment 27%; Lake area 22%; Tranquility 11%; City/urban area 10%; and Activity/leisure 9% (swimming, walking, biking, outdoor life).

### Regression Analyses

As can be seen in Table 1, a significant relationship between personal and collective identity and personal well-being showed that psychological mechanisms of people-place bonding accounted for 33% of variance in well-being. However, and in line with Hypothesis 1, the link between *personal* identity and *personal* well-being was stronger than between *collective* identity and *personal* well-being (see β statistics in Table 1, 0.40 vs. 0.24, indicating the slope of the regression lines). Thus, the better the personal/collective congruency between the phenomena of identity and well-being the higher well-being will be perceived at the favorite place.

**TABLE 1:** Regression statistics [standardized coefficients Beta (β)] for the relationships between predictors Personal Identity (PI) and Collective identity (CI) and the criterion variable *Personal* Well-being.

*R*^2^	Beta	*SE*	df	MS	*F*	*t*	Significance
0.33			2,675	129.1	167.02		0.00
	0.40 (PI)	0.03				10.30	0.00
	0.24 (CI)	0.03				6.15	0.00

Similar to the above, it was shown that psychological mechanisms of people-place bonding accounted for 27% of variance in well-being (see Table 2). However, and in line with Hypothesis 2, the link between *personal* identity and *collective* well-being was weaker than between *collective* identity and *collective* well-being (β statistics 0.21 vs. 037. This mirrors and replicates the above results suggesting that people perceive highest place-related well-being when the phenomena of place-related identity and well-being match (compare Tables 1, 2).

**TABLE 2:** Regression statistics [standardized coefficients Beta (β)] for the relationships between predictors Personal Identity (PI) and Collective Identity (CI) and the criterion variable *Collective* Well-being.

*R*^2^	Beta	*SE*	df	MS	*F*	*t*	Significance
0.27			2,654	110.77	122.32		0.00
	0.21 (PI)	0.03				5.16	0.00
	0.37 (CI)	0.03				9.06	0.00

In line with Hypotheses 2 and 4, significant relationships between personal and collective identity and personal willingness to sacrifice showed that psychological mechanisms of people-place bonding accounted for 16% of variance in willingness to sacrifice (see Table 3). However, and in line with Hypothesis 5, the link between *personal* identity and *personal* willingness to sacrifice well-being was slightly weaker than between *collective* identity and *personal* willingness to sacrifice (Table 3, β statistics 0.19 vs. 026. As predicted, this indicates an advantage of collective over individual commitment (measured as place-bonding) in willingness to sacrifice.

**TABLE 3:** Regression statistics [standardized coefficients Beta (β)] for the relationships between predictors Personal Identity (PI) and Collective Identity (CI) and the criterion variable *Personal* Willingness to Sacrifice.

*R*^2^	Beta	*SE*	df	MS	*F*	*t*	Significance
0.16			2,653	173.65	63.32		0.00
	0.19 (PI)	0.05				4.22	0.00
	0.26 (CI)	0.06				5.82	0.00

Additionally, as can be seen in Table 4, the link between *personal* identity and *collective* willingness to sacrifice was much weaker than between *collective* identity and *personal* willingness to sacrifice (β statistics 0.09 vs. 0.37). This underlines that the above obtained results suggest an advantage of collective vs. individual commitment (measured as place-bonding) in willingness to sacrifice, both personal and collective.

**TABLE 4:** Regression statistics [standardized coefficients Beta (β)] for the relationships between predictors Personal Identity (PI) and Collective Identity (CI) and criterion variable *Collective* Willingness to Sacrifice.

*R*^2^	Beta	*SE*	df	MS	*F*	*t*	Significance
0.16			2,648	179.03	71.71		0.00
	0.09 (PI)	0.05				2.10	0.04
	0.37 (CI)	0.06				8.34	0.00

### Mediation Analyses

**FIGURE 2:**
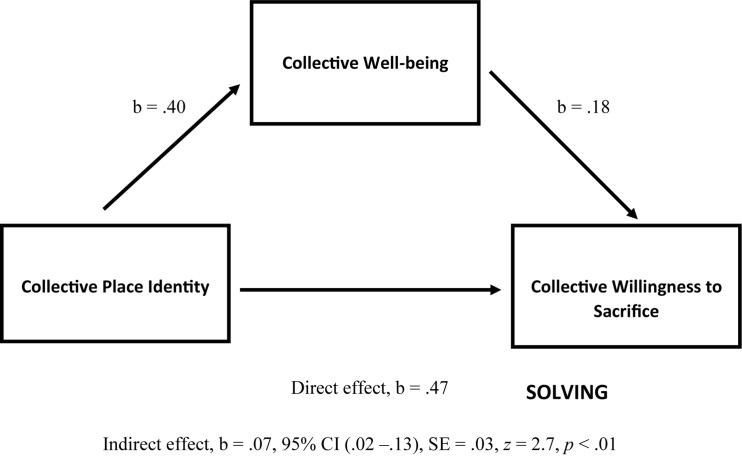
Mediation model of Personal Place Identity (PI) as a predictor of Personal Willingness to Sacrifice (PS), mediated by Personal Well-being (PW), including the mediation analysis statistics for relationships of: (1) PI → PW; (2) PW → PS; (3) PI → PS (direct effect); and (4) PI → PS via PW (indirect effect = mediation) (b is an unstandardized regression coefficient, and CI is confidence interval for the bootstrap methods, between BootLLCI and BootULCI).

As proposed by Hypothesis 6, a mediation analysis, PROCESS, developed by Andrew F. Hayes (Hayes, 2013) for IBM SPSS was performed. As can be seen in Figure 2, the results showed that: (1) personal identity predicts personal well-being (*b* = 0.37, *p* < 0.001); (2) personal well-being predicts personal willingness to sacrifice (*b* = 0.41, *p* < 0.001); and (3) personal identity predicts personal willingness to sacrifice (*b* = 0.28, *p* < 0.001). It was also reported that personal identity as predictor of personal willingness to sacrifice (“direct effect”) was mediated (“indirect effect”) by personal well-being (*b* = 0.15, CI 0.10–0.20, *SE* = 0.03, *z* = 5.76, *p* < 0.001). The mediation test was performed by computing confidence intervals for the “indirect” effect using bootstrap methods. Concerning the effect size, all (un)standardized confidence intervals contained no-zero point estimates; thus, “we can be confident that the true effects’ size is different from no effect” (Field, 2013, p. 416).

**FIGURE 3:**
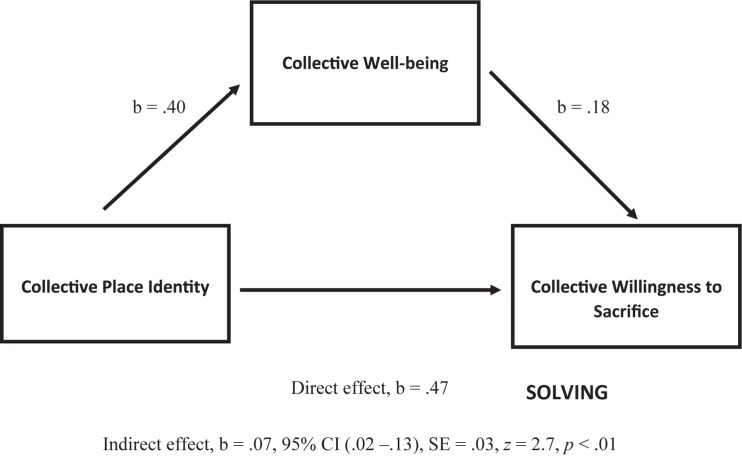
Mediation model of Collective Place Identity (CI) as a predictor of Collective Willingness to Sacrifice (CS), mediated by Collective Well-being (CW), including the mediation analysis statistics for relationships of: (1) CI → CW; (2) CW → CS; (3) CI → CS (direct effect); and (4) CI → CS via CW (indirect effect = mediation) (b is an unstandardized regression coefficient, and CI is confidence interval for the bootstrap methods, between BootLLCI and BootULCI).

In line with the above, and as can be seen in Figure 3, it was reported that: (1) collective identity predicts collective well-being (*b* = 0.40, *p* < 0.001); (2) collective well-being predicts collective willingness to sacrifice (*b* = 0.18, *p* < 0.001); and (3) collective identity predicts collective willingness to sacrifice (*b* = 0.47, *p* < 0.001). It was also shown that collective identity as a predictor of collective willingness to sacrifice (“direct effect”) was mediated (“indirect effect”) by collective well-being (*b* = 0.07, CI 0.02–0.13, *SE* = 0.03, *z* = 2.7, *p* < 0.01).

## Discussion

This study investigated relationships between place-related phenomena of identity, well-being and pro-environmental behavior (willingness to sacrifice) related to personal and collective favorite places of residents living in three Swedish municipalities, where the Lake Vänern Archipelago Biosphere Reserve is located (see Figure 1). We predicted that *four* of five positive associations would be stronger when they appeared congruently, and that *one* incongruent relationship would be stronger than the comparable congruent one (Hypotheses 1–5). Additionally, we predicted that the positive link between identity and willingness to sacrifice would be mediated by well-being (Hypothesis 6).

According to the qualitative data results, residents’ personal and collective favorite places were mostly categorized as nature-related sites (archipelago, forest, and agricultural landscape). Collective favorite places included, however, also the category of built-up environment. The most valued attributes ascribed to both types of places were related to nature, nature-related esthetics, tranquility, and social activities. This is in line with previous research suggesting that people are active in visiting and using favorite, nature-related places for emotional self-regulation, aesthetic, and social values (e.g., Korpela, 1989; Korpela and Hartig, 1996; Hammitt et al., 2006; Knez, 2006; Brown and Raymond, 2007; Knez and Eliasson, 2017; Knez et al., 2018b).

In line with previous findings (e.g., Knez and Eliasson, 2017; Knez et al., 2018a, b), a positive relationship between identity and well-being was reported, indicating that the stronger identity participants ascribed to a place the more well-being they felt visiting that site. As hypothesized, this association was shown to be stronger in congruent, compared to incongruent, relationships of: (1) *personal* identity and *personal* well-being related to a *personal* favorite place; and (2) *collective* identity and *collective* well-being related to a *collective* favorite place. This is consonant with previous studies showing that nature-related engagement and bonding may generate health-related benefits (Pretty, 2004; Pretty et al., 2005) of healthy-nature-healthy-people links (Maller et al., 2005; Carrus et al., 2015; Knez et al., 2018b). However, as far as we know, no previous research has indicated the importance of personal/collective *congruence* in nature-related bonding and well-being relationships.

Consonant, in general terms, with Davis et al. (2011; see also Iwata, 2002; Chen and Zheng, 2016), findings of a positive relationship between commitment (measured with a place identity instrument involving both emotional –attachment- and cognitive dimensions) to nature-related places and pro-environmental behavior of willingness to sacrifice, we reported that collective compared to personal place-identity predicts a stronger relationship with willingness to sacrifice to both personal and collective favorite places. In accordance with social identity theory (Tajfel, 1978; Tajfel and Turner, 1986), and previous research tentatively indicating an advantage of collective over individual commitment (e.g., Brunsting and Postmes, 2002; Clayton, 2003; Opotow and Brook, 2003; Dono et al., 2010), this suggests that a *group* compared to an *individual* is more willing to pay higher taxes and prices, as well as accept cuts in the standard of living, in order to maintain both types of favorite places; however, mostly for the sake of collective favorite sites.

Finally, and in accordance with, for example, the findings of Brown and Kasser (2005) and Davis et al. (2011), the mediation analyses showed that the relationships between personal and collective identity and willingness to sacrifice was partly accounted for by well-being, in both types of place. This suggests that a curative effect of revisiting and staying in a personal/collective favorite place (Knez and Eliasson, 2017; Knez et al., 2018b) may significantly influence the pro-environmental behavior of willingness to sacrifice. People will, in other words, maintain and preserve (pro-environmental behavior) beloved sites, not only because they like them and think about them (place-identity), but also because they feel good (well-being) staying in and revisiting these places.

## Conclusion

Overall, our results suggest that: (1) The more we identify ourselves emotionally and cognitively with a place the more we will experience, in congruence with personal/collective bonding, well-being in that favorite place (In other words, I feel best visiting my favorite places, and I, as an individual belonging to a group, feel best visiting our favorite places); (2) Additionally, the curative feelings ascribed to a favorite place will make us more pro-environmental toward that place; that is, in order to maintain and preserve the beloved site, we will be more willing to pay higher taxes and prices, and accept cuts in our standard of living; (3) Pro-environmental behavior, however, is more related to us as a collective than as an individual, and more to our collective than personal favorite sites.

## Data Availability Statement

The datasets generated for this study are available on request to the corresponding author.

## Ethics Statement

The studies involving human participants were reviewed and approved by the University of Gothenburg. The patients/participants provided their written informed consent to participate in this study.

## Author Contributions

IK: most contribution. IE and EG: participated in writing the Materials and Methods section and checking the manuscript.

## Conflict of Interest

The authors declare that the research was conducted in the absence of any commercial or financial relationships that could be construed as a potential conflict of interest.
